# Comparison Is the Thief of Joy? Introducing the Attitudes Towards Social Comparison Inventory

**DOI:** 10.1177/10731911231203968

**Published:** 2023-10-24

**Authors:** Pascal Schlechter, Thomas Meyer, Nexhmedin Morina

**Affiliations:** 1 University of Cambridge, UK; 2 University of Münster, Germany

**Keywords:** social comparison, attitudes, self-motives, comparison theory

## Abstract

Social comparison has a significant impact on individuals’ motivation, affect, and behavior. However, we lack a scale that captures individual differences in attitudes toward social comparison. To address this gap, we developed the Attitudes Toward Social Comparison Inventory (ASCI) drawing on existing scales that tap into metacognitive beliefs about worrying, self-motives, beliefs about emotions, and the general comparative-processing model. We examined the psychometric properties of the ASCI in a longitudinal study (*N* = 1,084), and a second (*N* = 550) and third cross-sectional study (*N* = 306). Through exploratory and confirmatory factor analyses, we identified a 12-item two-factor solution capturing positive and negative attitudes toward social comparison. The ASCI demonstrated measurement invariance across gender and time. The two factors were differentially and longitudinally associated with relevant constructs, including social comparison, metacognitive beliefs about worrying, depression, self-concept clarity, envy, and self-esteem. The ASCI facilitates comprehensive investigations of social comparison processes.

Social comparison—defined as comparing oneself with others—shapes people’s judgment, motivation, affect, and behavior (Baldwin & Mussweiler, 2018; Festinger, 1954), and serves motives of self-evaluation, namely, self-enhancement, self-verification, self-assessment, and self-improvement (Gregg et al., 2011; Morina, 2021; Sedikides & Strube, 1997). Social comparison outcomes can be broadly categorized as upward (e.g., perceiving oneself as less good looking than one’s best friend), lateral (e.g., perceiving oneself as equally good looking as one’s neighbor), and downward (e.g., perceiving oneself as better looking than one’s brother). Accordingly, the outcome of social comparison can be perceived as aversive (i.e., threatening the motives of the comparer), neutral, or appetitive (i.e., consonant with or challenging the motives of the comparer).

Due to the ubiquitous role of social comparison in self-evaluation and behavior, it is conceivable that individuals develop different attitudes toward social comparison. However, to date, interindividual differences in attitudes toward social comparison have been underinvestigated. A better understanding of metacognitive processes may help elucidate how and why individuals choose to engage in social comparison (Festinger, 1954). While the need to serve self-motives may underlie positive attitudes that encourage individuals to engage in social comparison (Gregg et al., 2011), individuals may hold negative attitudes toward social comparison as it often produces negative cognitive and emotional reactions (Morina & Schlechter, 2023). To facilitate a more in-depth understanding of these processes, the present paper, therefore, reports the development and validation of the Attitudes Toward Social Comparison Inventory (ASCI), which captures individuals’ positive and negative attitudes toward social comparison.

## Positive Beliefs About Social Comparison

There are several reasons why individuals may hold positive views about social comparison. In general, social comparison informs individuals about one's self-attributes, as all self-judgments essentially rely on frames of references, including social ones (Morina, 2021; Vlaev et al., 2011). For instance, when individuals construe judgments about how they are doing academically, they may rely on their academic peers as a frame of reference (Meyer et al., 2022; Unkelbach et al., 2023). Therefore, social comparison serves humans’ innate self-motives (Gerber et al., 2018; Morina, 2021; Unkelbach et al., 2023), four of which have been described extensively in the literature: self-enhancement, self-verification, self-assessment, and self-improvement (Sedikides & Strube, 1997). Depending on these motives and their activation across contexts, individuals may hold different positive beliefs about social comparison. For example, individuals with high levels of self-assessment (i.e., striving to know the truth about the self) may view social comparison positively because it informs them about where they stand socially. Moreover, by means of social comparison, individuals can become aware of their weaknesses and strengths, gain more accurate self-knowledge and a better intuition to guide them in important life decisions. For similar reasons, individuals scoring high on self-verification (i.e., striving to confirm preexisting view of oneself) may hold positive attitudes toward social comparison, as they may selectively and deliberately engage in comparison with standards that fit into their existing self-concept (Gregg et al., 2011). Meanwhile, individuals with elevated levels of the self-enhancement motive (i.e., striving to view oneself positively) may selectively and deliberately engage in downward social comparison as a means of enhancing their self-esteem or to feel better (Gregg et al., 2011). They may selectively compare themselves with others they consider to be worse-off and avoid comparisons with better-off individuals. Consequently, they may hold positive attitudes toward social comparison as they help them feel better about themselves. Finally, individuals scoring high on self-improvement (i.e., striving to improve oneself) may hold positive views toward social comparison (e.g., to very skilled or successful people), as these can guide them in improving their skills or abilities (Gregg et al., 2011). To that end, they tend to frequently compare themselves with individuals who are better-off on some relevant comparison dimension. Such upward comparisons are likely to produce positive behavioral outcomes given that the desired end state is perceived as attainable (Morina, 2021). For instance, an individual may compare with a friend who is doing better academically. By realizing that her friend engages in healthier sleep patterns and a productive learning routine, she can adjust her own schedule and establish similar routines. Therefore, these individuals may be more likely to hold positive attitudes toward social comparison as they may help improve themselves. Yet, these proposed mechanisms may be influenced by the context-dependent activation of self-motives, which remains to be investigated empirically (Morina, 2021), reiterating the need for a measurement of attitudes toward social comparison.

## Negative Beliefs About Social Comparison

People are likely to also hold negative attitudes toward social comparison. Individuals often communicate to each other that comparing with other people is bad, leading to normative standards that one should not compare with other individuals (e.g., Nolte, 2020). In addition, comparison appraised as threatening the motives of the comparer often elicits negative cognitive, emotional, and behavioral outcomes (Morina & Schlechter, 2023). Indeed, literature suggests that negative self-evaluations relative to others are associated with more symptoms of depression and anxiety (McCarthy & Morina, 2020). Accordingly, individuals may believe that social comparison is fundamentally bad for them and deliberate engagement in comparison may harm them psychologically. Such attitudes would be similar to (and possibly associated with) negative attitudes toward emotions (e.g., believing that having an emotion is harmful, damaging, or uncontrollable; Becerra et al., 2020; Karnaze & Levine, 2020; Manser et al., 2012). In addition, holding negative attitudes toward social comparison may moderate both the frequency and the affective impact of such comparisons. For instance, when individuals think of comparison as uncontrollable or harmful, noticing them may become highly distressing and instigate a cascade of maladaptive responses, such as thought suppression, rumination, or repetitive engagement in additional comparisons to ameliorate the negative self-evaluation (e.g., secondary or tertiary comparisons; see Morina, 2021). As such, negative attitudes toward comparison may share similarities to maladaptive metacognitions in the context of excessive worrying (Wells & Carter, 2001). The metacognitive model of pathological worrying suggests that worrying constitutes an adaptive process up to a certain degree, but can become excessive and maladaptive, in particular when individuals appraise their thought processes negatively (cf. type 2 worries; Wells & Cartwright-Hatton, 2004). Taken together, negative attitudes toward social comparison may play an important role in the habitual frequency with which individuals engage in them, as well as in the distress caused by aversive comparison, potentially contributing to overwhelming feelings of helplessness, loss of control, increased worry or rumination (Schlechter & Morina, 2023), lower self-efficacy, or negative self-views (e.g., “Not being able to stop comparing with others means that I am weak or indecent”).

## Existing Measurement Approaches

From a comparative-processing perspective, attitudes toward social comparison may be essential both in determining when and why individuals engage in social comparison, and in shaping the engendered cognitive, emotional, and behavioral reactions (Morina, 2021). Understanding attitudes toward social comparison would also enable us to address myriad research gaps (for review, see Morina, 2021), including the question if and when attitudes about social comparison transits from being adaptive (e.g., “Comparing informs me about what I can and cannot do”) to becoming maladaptive (e.g., “I cannot control my comparison to others”). In general, a wide range of instruments and paradigms have been developed to study social comparison (Gerber et al., 2018). Current measurement approaches focus on different ways to assess social comparison, for instance, by assessing individuals’ perceived social rank (Allan & Gilbert, 1995), or social comparison in specific domains such as physical appearance (Schaefer & Thompson, 2018) or well-being (Morina & Schlechter, 2023). However, to the best of our knowledge, there is currently no measure that taps into beliefs about social comparison, which constitutes an important gap in current literature on social comparison. Therefore, we developed the ASCI in the present study taking inspiration from scales that assess attitudes toward cognitions and emotions. To this end, we examined different questionnaires tapping into metacognitions concerning worrying (Wells & Cartwright-Hatton, 2004), self-evaluation motives (Gregg et al., 2011), and beliefs about emotions (Becerra et al., 2020; Karnaze & Levine, 2020; Manser et al., 2012), as a starting point to developing items.

The Meta-Cognitions Questionnaire–30 (MCQ-30) is pertinent because it assesses metacognitions about worrying (Wells & Cartwright-Hatton, 2004) and their crucial role in psychopathology (Wells & King, 2006). Similarly, understanding metacognitive aspects of comparative behavior may enable a greater understanding of (mal)adaptive beliefs about social comparison. In addition, item development was inspired by a set of questions capturing the self-evaluation motives self-enhancement, self-verification, self-assessment, and self-improvement presented by Gregg et al. (2011), as these motives constitute the major reasons to derive value from comparison outcomes. Consequently, these motives may shape how positively individuals think about comparison and how often they deliberately engage in comparison (Morina et al., 2022). A further set of relevant instruments stems from research concerning beliefs about emotions, which can be perceived as help or hindrance for one’s general psychological functioning. Higher endorsement of perceiving emotions as help (vs. hindrance) predicted the use of reappraisal strategies, which in turn predicted greater happiness (Karnaze & Levine, 2020). Other scholars have proposed similar differentiations concerning beliefs and metacognitions about emotions, such that emotions may be perceived as either useful or overwhelming, uncontrollable and potentially harmful (Becerra et al., 2020; Manser et al., 2012). In view of the conceptual overlap in these scales with our considerations on social comparison, we took direct inspiration from these instruments in constructing our scale (Table 1).

**Table 1.** Item Development.

Item	Valence	Motive	Attitude/belief	Source/ informed by
1. It is never rational for me to compare	Negative	—	Usefulness	EBQ (Becerra et al., 2020)
2. Comparing informs me about myself	Positive	Assessment	Usefulness	gComp (Morina, 2021)
3. Comparing is bad for me	Negative	—	Controllability	MCQ-30 (Wells & Cartwright-Hatton, 2004)
4. Comparing helps me discover that I have good qualities	Positive	Enhancement	Usefulness	SMS (Gregg et al., 2011)
5. Comparing prevents me from focusing on what matters most	Negative	—	Controllability	gComp (Morina, 2021)
6. Comparing helps me improve myself	Positive	Improvement	Usefulness	SMS (Gregg et al., 2011)
7. There is very little use for comparing myself to others	Negative	—	Usefulness	BEQ (Manser et al., 2012)
8. Comparing helps me reassure myself that I am the type of person I think I am	Positive	Verification	Usefulness	SMS (Gregg et al., 2011)
9. Comparing makes me unhappy	Negative	—	Controllability	gComp (Morina, 2021)
10. Comparing informs me about what I can and cannot do	Positive	Assessment	Usefulness	gComp (Morina, 2021)
11. I cannot control my comparisons to others	Negative	—	Controllability	MCQ-30 (Wells & Cartwright-Hatton, 2004)
12. Comparing helps me feel better about myself	Positive	Enhancement	Controllability	SMS (Gregg et al., 2011)
13. Comparing is usually a waste of time for me	Negative	—	Usefulness	HHTEM (Karnaze & Levine, 2020)
14. Comparing helps me get things clearer in my mind	Positive	Improvement	Usefulness	MCQ-30 (Wells & Cartwright-Hatton, 2004)
15. Comparing makes me realize my shortcomings	Negative	—	Controllability	HHTEM (Karnaze & Levine, 2020)
16. Comparing reminds me who I am	Positive	Assessment	Usefulness	gComp (Morina, 2021)
17. I constantly compare myself to others, even though I try not to	Negative	—	Controllability	MCQ-30 (Wells & Cartwright-Hatton, 2004)
18. Comparing helps me know where I stand socially	Positive	Assessment	Usefulness	gComp (Morina, 2021)
19. I try not to let comparisons guide me	Negative	—	Usefulness	HHTEM (Karnaze & Levine, 2020)
20. Comparing helps me avoid problems in the future	Positive	Improvement	Usefulness	MCQ-30 (Wells & Cartwright-Hatton, 2004)
21. Comparing might harm me psychologically	Negative	—	Controllability	BEQ (Manser et al., 2012)
22. Comparing helps me know where I stand in society	Positive	Assessment	Usefulness	gComp (Morina, 2021)
23. Comparing stops me from getting things done	Negative	Improvement	Controllability	BEQ (Manser et al., 2012)
24. Comparing helps me make decisions about what to do next	Positive	Improvement	Usefulness	BEQ (Manser et al., 2012)

*Note.* EBQ = Emotion Beliefs Questionnaire; gComp = general comparative-processing model; MCQ-30 = Meta-Cognitions Questionnaire–30; SMS = Self-motives Scale; BEQ = Beliefs about Emotions Questionnaire; HHTEM = Help and Hinder Theories about Emotion Measure.

## The Present Study

To develop and validate the ASCI, we broadly defined positive and negative attitudes, and included any attitudes that individuals may have concerning social comparison. We then examined the psychometric properties of our scale in three studies. Following rational, expert-based item development in Study 1, we employed a data-driven approach and explored the underlying structure of the ASCI. Despite this data-driven procedure, we expected to find two main factors reflecting positive and negative attitudes toward social comparison. The structure of the scale was then retested with confirmatory approaches in a subsample of Study 1 and a 3-month follow-up. We conducted a second and third study to confirm the structure and validity of the English and German version of the ASCI. To test the validity of the ASCI, we examined its correlations with different constructs. Given that the ASCI is designed to measure attitudes toward social comparison, we anticipated that the resulting factors would display significant relationships with social comparison (Allan & Gilbert, 1995). Moreover, given the conceptual parallels between social comparison and comparison with other standards (for review, see Morina, 2021), such as one’s own past (i.e., past temporal), certain expectations (i.e., criterion-based), other life domains (i.e., dimensional), or alternative scenarios (i.e., counterfactual), we predicted that the ASCI would also be associated with the frequency of engaging in these types of comparison. We also expected moderate correlations with metacognitions related to worry, due to their similar cognitive underpinnings (Wells & Cartwright-Hatton, 2004). In view of the established link between social comparison and various mental health and well-being outcomes, including depression, anxiety, life orientation (McCarthy & Morina, 2020), or self-esteem (Unkelbach et al., 2023), we anticipated that ASCI scores would demonstrate associations with these variables as well. Due to their proposed role in the motivation to engage in social comparison, positive attitudes measured with the ASCI were expected to correlate positively with self-evaluation motives (Gregg et al., 2011). In addition, we expected that positive and negative attitudes would be positively and negatively associated with the perceived controllability and usefulness of emotions, respectively (Becerra et al., 2020). We expected especially negative attitudes to be related to lower levels of self-concept clarity (Campbell et al., 1996). Positive attitudes toward social comparison were expected to be associated with benign envy and negative attitudes toward social comparison with malicious envy (Lange & Crusius, 2015). In general, we anticipated that the emerging factors (if more than one factor was found) would display differential associations, such that positive attitudes would be more strongly associated with positive outcomes and less strongly with negative outcomes, while negative attitudes would be more strongly associated with negative outcomes (cf. supra).

## Method

### Participants and Design

We report how we determined our sample size, all data exclusions, all manipulations, and all measures in the study. In Study 1, we aimed to have a sample size of at least 1,000 participants, which is recommended for scale development, as this allows adequately low measurement errors and stable factor loadings (Boateng et al., 2018). The sample sizes for the other two studies were based on practical considerations of available resources for participant compensation. In Study 1, *N* = 1,119 participants provided informed consent. They were recruited from online panel provider Prolific Researcher (Palan & Schitter, 2018). Participants had to be fluent in English and older than 17 years. We excluded participants who responded to our survey in less than 10 min as this response time was deemed too fast for conscientious responses (*n* = 21). Likewise, we excluded participants with a response time of over 1 hr, as these participants may have stopped the survey in-between (*n* = 12). Moreover, we examined response variability to reveal unreasonable response patterns before we recoded negatively phrased items. Two participants had no variance on the ASCI items, although some items were reversely coded. As this may indicate an unrealistic response pattern, we excluded these participants. Accordingly, *N* = 1,084 participants were considered for final analysis in the first assessment in Study 1. Participants were on average 31.56 (*SD* = 10.49; age range: 18–75) years old and 39.6% (*n* = 429) of them were female, while 12 participants did not indicate their gender. Most participants were single (*n* = 678) or married/in a domestic partnership (*n* = 371). Participants had less than a high school degree (*n* = 11), a high school degree or equivalent (*n* = 198), some college education but no degree (*n* = 204), an associate degree (*n* = 51), a bachelor’s degree (*n* = 417) or a graduate degree (*n* = 203). When asked how they estimate their social standing on a ladder from 0 to 10, participants indicated 5.48 (*SD* = 1.56) on average. We conducted a follow-up assessment 3 months later to test whether the psychometric properties remain similar across time and to estimate the stability of the obtained scores. In the follow-up, *N* = 948 of the initial participants participated, of which six had to be excluded because of no variance in their ratings on positive and negatively phrased items before items were recoded, leading to *N* = 942 participants (86.9% of the initial sample). Individuals who did not participate in the second assessment were slightly younger (*M* = 29.29, *SD* = 9.55) than those who participated at both time points (*M* = 31.95, *SD* = 10.57), *t*(276.03) = 3.36, *p* < .01.

In Study 2, we assessed another English-speaking sample, again recruited from Prolific, consisting of *N* = 550 participants. Participants were on average 33.97 (*SD* = 11.54; age range: 18–72) years old and 32.2% (*n* = 177) were female, while seven participants did not indicate their gender. Most participants had a bachelor’s degree (*n* = 234) and were either single (*n* = 331) or married (*n* = 202).

In Study 3, we recruited a German sample of *N* = 306 participants. Participants were recruited online via social media channels and different newsletters. To be eligible, participants were required to possess fluency in German and exceed the age of 17 years. The majority of the sample were female (*n* = 232; 75.8%), while two participants did not indicate their gender. The sample was on average 24.97 (*SD* = 5.93; age range: 19–70) years old. Most participants had a high school (*n* = 148) or university degree (*n* = 152). We did not detect any suspicious response patterns in the samples of Study 2 and Study 3; hence, no data were excluded. All studies received ethical approval from the University of Münster.

### ASCI Scale Development

The aim of the ASCI development was to capture positive and negative attitudes toward social comparison. We focused on positive and negative attitudes toward social comparison, as we were specifically interested in how they influence social comparison behavior and other outcomes. Explicitly neutral attitudes were not included as we expected neutral attitudes to have limited incremental predictive value relative to attitudes that carry an evaluative valence. Attitudes were defined broadly and could cover any attitudes that individuals may hold concerning social comparison. As there was no clear starting point for such a scale in the literature, we based the item development process on existing scales tapping into adjacent constructs, that is, primarily metacognitive beliefs about worrying (Wells & Cartwright-Hatton, 2004), self-evaluation motives (Gregg et al., 2011), and beliefs about emotions (Becerra et al., 2020; Karnaze & Levine, 2020; Manser et al., 2012). After the development of candidate items, we broadly evaluated (a) whether the respective item clearly carries either positive or negative valence concerning social comparison; (b) for positive items, whether they align with one of the motives of self-enhancement, self-verification, self-assessment, or self-improvement; and (c) for all items, whether they fit in one of the categories usefulness or controllability (which reflect important dimensions on which metacognitions toward worries or emotions are evaluated; for example, Becerra et al., 2020). In the latter categorization, note that all items on the positive scale address some aspect of usefulness (e.g., “Comparing helps me improve myself”), whereas items on the negative scale could address either usefulness (e.g., “There is very little use for comparing myself to others”) or controllability (e.g., “Comparing stops me from getting things done”). This information can be found for each item in Table 1, which also describes the main source used to formulate the item or that informed item development. Along the mentioned scales, the general comparative-processing model (gComp) served as an overarching theoretical account for item development (Morina, 2021).

In a first step, we formulated items independently based on the outlined considerations. This led to an initial item pool of 49 items. Then, we discussed each of the items and evaluated their fit with the scope of the scale, clarity, and unambiguity of the language, and nonredundancy within the item pool. In several revision rounds, we eliminated items based on these criteria, which resulted in the final item pool of 24 items. These items were then sent to two different English native speakers who refined the wording in several revision rounds. The German items were developed using back translation procedures with several rounds of back-and-forth translations involving two native speakers of both languages. The final item pool of 24 items can be found in Table 1. For each item, respondents had to indicate their agreement on a 5-point Likert-type scale ranging from 1 (*strongly disagree*) to 5 (*strongly agree*) with a score of 3 indicating neutral attitudes toward social comparison. The instruction reads, “This questionnaire asks about *your beliefs about comparing to other people*. For each statement, please rate how much you agree or disagree that the statement is true in general.” The final ASCI can be found in the open science framework both in English and German https://osf.io/u9b4n/?view_only=c945a29e3e33449b937e176e5e30f3e0.

### Study 1: Validation Scales

#### Comparison Standards Scale for Well-Being

The Comparison Standards Scale for Well-Being (CSS-W; Morina & Schlechter, 2023) examines habitual upward and downward comparison with social, temporal, counterfactual, and criteria-based standards related to one’s own well-being. The CSS-W consists of (a) 14 obligatory items assessing the frequency of well-being comparisons in the past 3 weeks on 6-point Likert-type scales (0 = *not at all* to 5 = *very often*). When this question is answered with a score above 0, respondents are given (2) a respective subitem pertaining to the discrepancy compared with the standard on a 6-point Likert-type scale (0 = *not at all* to 5 = *much better/worse;* 14 potential subitems in total). A further subitem is then asked tapping into (3) the affective impact on a bipolar 7-point Likert-type scale for affective impact (–3 = *much worse* to +3 = *much better*). For instance, an upward social comparison item first asks about the frequency: “Over the past 3 weeks when considering your well-being, how often have you compared with others in your close circles who were doing better than you?” If participants indicate values higher than “0—*not at all*,” they are additionally asked, “How much better have you considered them to be doing?” (i.e., discrepancy assessment) and “On average during the past 3 weeks, how did the comparison make you feel?” (i.e., affect assessment). The structure of the scales is two-dimensional for all three components of comparison (frequency, discrepancy, and affect), capturing the two correlated latent factors: aversive and appetitive comparisons (Morina & Schlechter, 2023). This results in six total scores. *Upward* social, past temporal, counterfactual, and criteria-based comparisons, and *downward* prospective temporal comparisons constituted the aversive comparison factor (i.e., threatening one’s own motives). *Downward* social, past temporal, counterfactual, and criteria-based as well as *upward* prospective temporal comparisons constituted the appetitive comparison factor (i.e., consonant with or challenging the motives). We used resulting mean scores for aversive comparison frequency (α = .79), aversive comparison discrepancy (α = .73), aversive comparison affective impact (α = .76), appetitive comparison frequency (α = .73), appetitive comparison discrepancy (α = .69), and appetitive comparison affective impact (α = .63).

#### Social Comparison Scale

The Social Comparison Scale (SCS) uses a semantic differential methodology to assess social rank evaluations (Allan & Gilbert, 1995). Participants make global social comparison judgments of themselves relative to others with bipolar dimensions rated from 1 to 10. For example, the scale asks, “In relation to others I generally feel”: *Inferior* 1 2 3 4 5 6 7 8 9 10 *Superior*, with 11 such statements tapping into inferiority–superiority, attractiveness, and insider–outsider ratings. Mean scores were calculated as an index of how people rank themselves compared with others across a range of attributes. Internal consistency in our study was α = .93.

#### Meta-Cognitions Questionnaire–30

We used two subscales of the MCQ-30 (Wells & Cartwright-Hatton, 2004), namely, *positive beliefs about worry* and *beliefs about uncontrollability of thoughts and corresponding danger*. Items are scored on a 4-point scale (1 = *do not agree* to 4 = *agree very much*). Internal consistencies were α = .91 for both factors.

#### Life Orientation Test–Revised

The Life Orientation Test–Revised (LOT-R; Scheier & Carver, 1992) measures positive life orientation with seven items. An example item is, “In uncertain times, I usually expect the best.” Response options range from 1 (*strongly disagree*) to 7 (*strongly agree*). Internal consistency of the scale in the present study was α = .84.

#### Patient Health Questionnaire–4

Depression and anxiety symptoms (both αs = .84) were assessed with the Patient Health Questionnaire–4 (PHQ-4), a four-item questionnaire assessing symptoms on a 4-point scale from 0 (*not at all*) to 3 (*nearly every day*). Symptom endorsement of the last 2 weeks was assessed, concerning the core symptoms of depression and anxiety (loss of interest, depressed mood, anxiety, constant worries; Löwe et al., 2010). The PHQ-4 was also used in Study 2 (α = .85 for anxiety and α = .84 for depression).

#### Rosenberg Self-Esteem Scale

The Rosenberg Self-Esteem Scale (RSES; Rosenberg, 1965) was used to measure general self-esteem consisting of 10 items. Items (e.g., “On the whole, I am satisfied with myself”) are rated on a 4-point Likert-type scale (0 = *strongly disagree* to 3 = *strongly agree*). Cronbach’s alpha in this study was α = .92.

### Follow-Up: Validation Scales

In the follow-up, we also used the CSS-W (αs = .61–.79), MCQ-30 subscales (positive beliefs about worry, α = .91, and beliefs about uncontrollability of thoughts and corresponding danger, α = .91), the PHQ-4 (α = .87 for anxiety and α = .84 for depression), and the RSES (α = .93).

### Study 2: Validation Scales

#### Self-Motives Scale

The Self-Motives Scale (SMS; Gregg et al., 2011) assesses motives of self-enhancement (α = .66), self-verification (α = .63), self-assessment (α = .74), and self-improvement (α = .66). Two items assess each of the four self-motives on a 7-point Likert-type scale (1 = *strongly disagree* to 7 = *strongly agree*).

#### Comparison Standards Scale for Appearance

The Comparison Standards Scale for Appearance (CSS-A; Morina et al., 2023) mirrors the CSS-W described above in its structure, but focuses on one’s own appearance as the dimension of comparison. In addition to social, temporal, counterfactual, and criterion-based standards, it also assesses comparisons relative to dimensional standards (e.g., comparing one’s appearance with some other personal attribute that may stand out, or compensate for one’s appearance). Cronbach’s alpha ranged from .69 to .83 for the appetitive and aversive frequency, discrepancy, and affective impact subscales.

#### Emotion Beliefs Questionnaire

The Emotion Beliefs Questionnaire (EBQ; Becerra et al., 2020) was used to assess people’s beliefs about emotions, specifically their controllability and usefulness. The EBQ consists of 16 items utilizing an 8-point Likert-type scale (1 = *disagree* to 8 = *strongly agree*). Based on a strong overarching bifactor, we used a single total EBQ score (α = .86).

#### Help and Hinder Theories About Emotion Measure

The Help and Hinder Theories About Emotion Measure (HHTEM; Karnaze & Levine, 2020) was used to assess individuals’ beliefs about emotion. Two subscales assess whether they perceive emotions as help (α = .78) or hindrance (α = .78), with four items each on a scale from 1 (*almost never or never*) to 7 (*almost always or always*).

#### Self-Concept Clarity Scale

The Self-Concept Clarity Scale (SCCS; Campbell et al., 1996) was used to measure the stability, clarity, and confidence of self-beliefs. This scale contains 12 items (α = .92) on a 7-point Likert-type scale (1 = *strongly disagree* to 7 = *strongly agree*).

#### Benign and Malicious Envy Scale

Benign and Malicious Envy Scale (BeMaS; Lange & Crusius, 2015) assesses benign (α = .83) and malicious (α = .83) envy with five items each on a 6-point Likert-type scale (1 = *strongly disagree* to 6 = *strongly agree*).

### Study 3: Validation Scales

In the German sample in Study 3, we used the German version of the CSS-W (Morina, 2020, αs = .56–.77), the SCS (see Ascone et al., 2017 for the German version α = .89), the PHQ-4 (see Löwe et al., 2010 for the German version, α = .69 for anxiety and α =.81 for depression), and the RSES (Roth et al., 2008, for the German version, α = .90).

### Analysis Plan

The data, materials, and code to reproduce the present analyses are openly available on the open science framework: https://osf.io/u9b4n/?view_only=c945a29e3e33449b937e176e5e30f3e0. Our studies were not preregistered. All analyses were conducted in R version 4.01 (R Core Team, 2021). Our analysis was data-driven, and we aimed to reduce the number of items for the final scale. First, we conducted exploratory factor analysis (EFA) to explore the underlying number of latent factors. Second, we used confirmatory factor analysis (CFA) to confirm this factor structure. To this end, we randomly split the sample of Study 1 into one sample for the EFA and one sample for the CFA. In the follow-up sample of Study 1 as well as in Study 2 and Study 3, we conducted CFAs to confirm the factor solution found in Study 1.

### Exploratory Factor Analysis

To explore the underlying structure of our data, we used EFA. To discern the number of latent factors, we conducted parallel analysis and visually inspected the scree-plot (Fabrigar & Wegener, 2011). In line with Fabrigar and Wegener (2011), we additionally used the Velicer’s minimum average partial (MAP) test, the very simple structure (VSS), and the Kaiser criterion (eigenvalues above 1). As rotation method, we chose promax rotation (allowing factors to be correlated), as we assumed that the underlying factors would be correlated among each other. As data were ordinal, we ran the EFA with the lavaan package in *R* (Rosseel, 2012) to use the weighted least squares mean and variance adjusted (WLSMV) estimator (Asparouhov & Muthén, 2010). We included items in subsequent analyses when their factor loadings exceeded .50 and when they displayed no double loadings (i.e., substantial factor loadings on more than one factor, that is, a loading of .20 or above on a different factor than the primary factor). After excluding items that did not fit our criteria to be included in further analyses, we repeated the EFA with the remaining items.

### Confirmatory Factor Analysis

To confirm the factor structure that was found in the EFA, we used the second subsample of Study 1 to conduct a CFA with this factor structure. The same procedure was applied for the follow-up data of Study 1 and the samples in Study 2 and Study 3. For the CFA, items were again treated as ordinal and we used the WLSMV estimator (Asparouhov & Muthén, 2010). To evaluate model fit, we used the following criteria: Comparative fit index (CFI) and Tucker–Lewis index (TLI) values > 0.95 indicate good fit and values > 0.90 indicate acceptable fit; root mean square error of approximation (RMSEA) and standardized root mean square residuals (SRMR) values < .05 indicate good fit and values < .10 indicate acceptable fit (Hu & Bentler, 1999). The lavaan package in R was used (Rosseel, 2012).

### Measurement Invariance Across Gender, Time, and Language

For a new measure, it is important to demonstrate that any differences in the manifest variables between different groups and across time reflect true-score differences. Research on habitual comparison frequencies suggests that gender differences may play a role in comparison processes (Morina & Schlechter, 2023). We, therefore, additionally tested whether the ASCI measures the same underlying construct among males and females and across time. To this end, we conducted measurement invariance analysis between men and women and across time from Study 1 to follow-up (Chen et al., 2020). Following Svetina et al. (2020), increasingly constrained nested models were sequentially tested against each other. The respective constraints were added for each model in addition to the constraints introduced in the model before (Chen et al., 2020). First, the factor structure was constrained to be equivalent across gender and across the first assessment and the follow-up assessment (configural invariance/ baseline model). Second, we tested a model, in which the thresholds were constrained to be equal across groups or time (threshold invariance). Third, as recommended for ordinal data (Svetina et al., 2020; Wu & Estabrook, 2016) the factor loadings were additionally constrained to be equivalent to gauge whether the factor loadings and observed thresholds conditional on the latent factors do not differ across gender and time (threshold/loading invariance). Finally, the residual variances of the items were also constrained to be equal to scrutinize whether the amount of variance in the items not explained by the latent factors does not differ across gender and time (residual invariance; Chen et al., 2020). Longitudinal measurement invariance was modeled in a wide format, in which we allowed the residuals of the same indicators to freely vary over time (Liu et al., 2017). As data were ordinal, we used theta parameterization. To detect violations of measurement invariance, we evaluated changes (Δ) in the CFI and RMSEA. The differences between the fit indices of two nested models indicate that measurement invariance has been violated when ΔCFI exceeds 0.010 and ΔRMSEA exceeds 0.007 (Chen et al., 2020; Neufeld et al., 2023).

### Nomological Network

To examine the convergent validity of the ASCI, we calculated scale composite scores of the resulting scales and correlated them with the mean values of the variables that were theoretically expected to be associated with our new measure (Table 7). Specifically, in Study 1 and its follow-up, the resulting scores were correlated with aversive and appetitive well-being comparison frequency, discrepancy, and affective impact as well as social comparison, metacognitive beliefs about worry (positive beliefs & beliefs about uncontrollability and danger), life orientation, depression, anxiety, and self-esteem. In Study 2, we correlated the corresponding scores with self-motives, aversive and appetitive appearance-related comparisons, beliefs about emotions, self-concept clarity and benign and malicious envy in the English-speaking sample. In Study 3, scores were correlated with comparison frequency, discrepancy and affective impact, social comparison, depression, anxiety, and self-esteem.

## Results

### Descriptive Statistics and Exploratory Factor Analysis

Descriptive statistics for the initial 24 items can be found in Table 2. Skewness and kurtosis for all items were good. Next, we conducted the EFA with the first subsample of Study 1 (*N* = 542). The scree-plot and the Kaiser criterion clearly suggested a two-factor solution. The parallel analysis and MAP suggested three underlying latent factors, whereas the VSS indicated a one-factor solution. Given that the eigenvalue of the third factor was 0.65 and thus below the Kaiser criterion, we decided to continue with a two-factor solution as the third factor did not explain substantial variance.

**Table 2.** Descriptive Statistics of All Items.

Item	Study 1 (*N* = 1,084)				Follow-up (*N* = 942)				Study 2 English (*N* = 550)				Study 2 German (*N* = 306)			
	*M*	*SD*	*Sk*	*Kt*	*M*	*SD*	*Sk*	*Kt*	*M*	*SD*	*Sk*	*Kt*	*M*	*SD*	*Sk*	*Kt*
1. It is never rational for me to compare	2.82	1.01	0.23	−0.67	—	—	—	—	—	—	—	—	—	—	—	—
2. Comparing informs me about myself	3.33	0.93	−0.62	−0.33	3.22	1.04	−0.64	−0.58	3.42	0.93	−0.68	−0.01	3.34	0.99	−0.67	−0.32
3. Comparing is bad for me	3.45	1.09	−0.36	−0.69	3.34	1.18	−0.26	−0.83	3.09	1.24	0.04	−1.09	3.36	1.08	0.34	−0.59
4. Comparing helps me discover that I have good qualities	3.12	1.02	−0.25	−0.74	—	—	—	—	—	—	—	—	—	—	—	—
5. Comparing prevents me from focusing on what matters most	3.24	1.11	−0.26	−0.74	3.14	1.18	−0.20	−0.93	3.01	1.20	0.06	−1.02	3.11	1.17	−0.22	−1.00
6. Comparing helps me improve myself	3.05	1.11	−0.14	−0.95	—	—	—	—	—	—	—	—	—	—	—	—
7. There is very little use for comparing myself to others	3.13	1.15	−0.06	−1.01	—	—	—	—	—	—	—	—	—	—	—	—
8. Comparing helps me reassure myself that I am the type of person I think I am	2.94	1.02	−0.13	−0.72	2.97	1.12	−0.26	−0.95	3.31	1.02	−0.50	−0.43	3.22	1.03	−0.38	−0.67
9. Comparing makes me unhappy	3.39	1.14	−0.44	−0.61	3.39	1.20	−0.40	−0.75	3.15	1.24	−0.12	−1.01	3.39	1.06	−0.41	−0.45
10. Comparing informs me about what I can and cannot do	3.20	1.04	−0.33	−0.59	3.12	1.13	−0.30	−0.95	3.25	1.10	−0.28	−0.73	3.62	1.07	−0.83	0.01
11. I cannot control my comparisons to others	2.96	1.24	−0.04	−1.17	—	—	—	—	—	—	—	—	—	—	—	—
12. Comparing helps me feel better about myself	2.46	1.03	0.32	−0.58	—	—	—	—	—	—	—	—	—	—	—	—
13. Comparing is usually a waste of time for me	3.22	1.14	−0.24	−0.90	—	—	—	—	—	—	—	—	—	—	—	—
14. Comparing helps me get things clearer in my mind	2.84	1.08	−0.02	−1.03	—	—	—	—	—	—	—	—	—	—	—	—
15. Comparing makes me realize my shortcomings	3.42	1.05	−0.63	−0.27	—	—	—	—	—	—	—	—	—	—	—	—
16. Comparing reminds me who I am	3.02	1.08	−0.19	−0.85	3.02	1.15	−0.26	−0.95	3.10	1.12	−0.25	−0.74	2.62	1.13	0.30	−0.77
17. I constantly compare myself to others, even though I try not to	3.12	1.26	−0.18	−1.13	—	—	—	—	—	—	—	—	—	—	—	—
18. Comparing helps me know where I stand socially	3.17	1.09	−0.40	−0.82	3.32	1.16	−0.59	−0.61	3.49	1.10	−0.63	−0.24	3.49	1.06	−0.59	−0.36
19. I try not to let comparisons guide me	3.53	1.00	−0.50	−0.36	—	—	—	—	—	—	—	—	—	—	—	—
20. Comparing helps me avoid problems in the future	2.70	1.03	0.21	−0.73	2.73	1.14	0.06	−1.02	2.94	1.13	−0.02	−0.82	2.75	1.13	0.20	−0.88
21. Comparing might harm me psychologically	3.53	1.16	−0.63	−0.49	3.27	1.20	−0.48	−0.75	3.28	1.23	−0.38	−085	3.68	1.11	−0.86	0.09
22. Comparing helps me know where I stand in society	3.09	1.08	−0.30	−0.80	—	—	—	—	—	—	—	—	—	—	—	—
23. Comparing stops me from getting things done	2.84	1.18	0.07	−1.02	2.84	1.25	0.04	−1.13	2.64	1.48	0.27	−0.86	2.49	1.16	0.40	−0.74
24. Comparing helps me make decisions about what to do next	2.82	1.05	0.01	−0.90	2.98	1.14	−0.29	1.06	3.19	1.11	−0.36	−0.74	2.74	1.14	0.36	−0.87

*Note. Sk* = skewness; *Kt* = Kurtosis.

### Item Selection

In a next step, we examined the factor loadings of the resulting two-factor solution (Table 3). First, we excluded items that loaded below our cut-off of .50, which only accounted for Items 1 and 19 (Table 3 for the item content). Next, we identified items with substantial double loadings (i.e., a factor loading of > .20 on a different factor than the primary factor), which was the case for Items 4, 6, 7, 11, 12, 13, 14, 15, and 17. A closer inspection of these items revealed that they were ambiguous and may be interpreted differently depending on contextual factors and the activation of different self-motives. This item exclusion resulted in a remaining item pool of 13 items. Accordingly, these items best represent the two factors underlying the data.

**Table 3.** Initial EFA Factor Loadings in the First Subsample of Study 1 (N = 542).

Item	Factor 1	Factor 2
1. It is never rational for me to compare	−.37	
2. Comparing informs me about myself	.65	—
3. Comparing is bad for me	—	.72
4. Comparing helps me discover that I have good qualities	.45	−.46
5. Comparing prevents me from focusing on what matters most	—	.51
6. Comparing helps me improve myself	.56	−.43
7. There is very little use for comparing myself to others	−.53	.34
8. Comparing helps me reassure myself that I am the type of person I think I am	.59	—
9. Comparing makes me unhappy	—	.86
10. Comparing informs me about what I can and cannot do	.67	—
11. I cannot control my comparisons to others	.45	.74
12. Comparing helps me feel better about myself	.42	−.47
13. Comparing is usually a waste of time for me	−.58	.38
14. Comparing helps me get things clearer in my mind	.69	−.26
15. Comparing makes me realize my shortcomings	.65	.32
16. Comparing reminds me who I am	.78	—
17. I constantly compare myself to others, even though I try not to	.51	.82
18. Comparing helps me know where I stand socially	.90	—
19. I try not to let comparisons guide me	−.43	—
20. Comparing helps me avoid problems in the future	.58	—
21. Comparing might harm me psychologically	—	.75
22. Comparing helps me know where I stand in society	.88	—
23. Comparing stops me from getting things done	—	.69
24. Comparing helps me make decisions about what to do next	.68	—

*Note*. Only double loadings >.20 are shown. EFA = exploratory factor analysis.

Inspection of the item content revealed that the first factor captures positive attitudes toward social comparison (eight items), whereas the second factor taps into negative attitudes toward social comparison (five items). Item 18 (“Comparing helps me know where I stand socially”) and Item 22 (“Comparing helps me know where I stand in society”) appeared to measure very similar contents, leading to potentially spurious item intercorrelations. Factor loadings of these items were also very similar (Table 3). Based on the consideration that Item 18 (“Comparing helps me know where I stand socially”) leaves greater room to refer to the proximal environment (e.g., friends, peers, and family) and aligns more closely with the putative underlying motives of engaging in social comparison, we decided to exclude Item 22. Accordingly, we had 12 items in our final item pool, of which Items 2, 8, 10, 16, 18, 20, and 24 loaded on Factor 1, and Items 3, 5, 9, 21, and 23 loaded on Factor 2 (Table 4 for the final item pool). Based on the item content, Factor 1 was coined *positive attitudes toward social comparison* and Factor 2 was coined *negative attitudes toward social comparison.* As robustness check, we examined whether a one-factor solution describes the data. Factor loading patterns were mixed with positive and negative factor loadings, reflecting positive and negative attitudes toward social comparison. Model fit was worse for the one-factor model (CFI = .89, TLI = .88, RMSEA = .20, SRMR = .15), compared with the two-factor model (CFI = .99, TLI = .99, RMSEA = .07, SRMR = .05). We conducted a second EFA with the selected items, which indicated a two-factor solution according to all criteria apart from the VSS, which suggested a one-factor solution. In the second EFA, all items had excellent factor loadings on their respective factors (Table 4 for the factor loadings of the second EFA). Model fit was good according to all criteria (CFI = .99, TLI = .99, RMSEA = .05, SRMR = .03).

**Table 4.** Standardized Factor Loadings of the Second EFA and of the Different CFAs.

Item	Second EFA (*N* = 542)		CFA Study 1 (*N* = 542)		CFA follow-up (*N* = 942)		CFA Study 2 (*N* = 550)		CFA Study 3 (*N* = 306)	
	F1	F2	F1	F2	F1	F2	F1	F2	F1	F2
1. Comparing informs me about myself	.65	—	.65	—	.83	—	.82	—	.69	—
2. Comparing helps me reassure myself that I am the type of person I think I am	.62	—	.71	—	.81	—	.79	—	.67	—
3. Comparing informs me about what I can and cannot do	.69	—	.61	—	.73	—	.62	—	.52	—
4. Comparing reminds me who I am	.85	—	.72	—	.81	—	.75	—	.56	—
5. Comparing helps me know where I stand socially	.77	—	.69	—	.73	—	.60	—	.62	—
6. Comparing helps me avoid problems in the future	.62	—	.70	—	.71	—	.70	—	.44	—
7. Comparing helps me make decisions about what to do next	.69	—	.70	—	.77	—	.70	—	.40	—
8. Comparing is bad for me	—	.76	—	.81	—	.82	—	.85	—	.84
9. Comparing prevents me from focusing on what matters most	—	.57	—	.64	—	.72	—	.77	—	.75
10. Comparing makes me unhappy	—	.89	—	.82	—	.85	—	.88	—	.90
11. Comparing might harm me psychologically	—	.79	—	.76	—	.83	—	.76	—	.79
12. Comparing stops me from getting things done	—	.72	—	.73	—	.79	—	.80	—	.64

*Note*. EFA = exploratory factor analysis; CFA = confirmatory factor analysis; F = Factor.

### Confirmatory Factor Analysis

We used the second subsample of Study 1 to conduct a CFA (*N* = 542) to confirm the two-factor solution, in which we allowed the two latent factors to covary among each other. Factor loadings of the items on their respective factor were good for all items (Table 4). As can be seen in Table 5, model fit was good according to the CFI and TLI and acceptable according to RMSEA and SRMR. The latent correlation among the two factors *positive attitudes* and *negative attitudes* was *r* = –.42. In the follow-up analysis of Study 1, all fit indices apart from the RMSEA indicated good or acceptable model fit for the two-factor solution. The correlation among the two latent factors was *r* = –.24.

**Table 5.** Model Fit for the Two-Factorial Solution in the CFAs.

Factor model	χ^2^(*df*)	*p*	CFI	RMSEA	SRMR	TLI	α Factor 1	α Factor 2	ω_total_ Factor 1	ω_total_ Factor 2
Two-factor model Study 1	229 (53)	<.001	.983	.078	.063	.978	.85	.83	.85	.83
Two-factor model follow-up	701 (53)	<.001	.980	.114	.080	.975	.89	.87	.89	.87
Two-factor model Study 2	310 (53)	<.001	.982	.096	.071	.977	.85	.88	.85	.88
Two-factor model Study 3	144 (53)	<.001	.978	.075	.073	.973	.71	.86	.71	.86

*Note.* CFA = confirmatory factor analysis; CFI = comparative fit index; RMSEA = root mean square error of approximation; SRMR = standardized root mean square residuals; TLI = Tucker–Lewis Index; *df* = degrees of freedom.

In both Study 2 and Study 3, model fit was good according to the CFI and TLI and acceptable according to RMSEA and SRMR. The latent correlation among the two latent factors *positive attitudes toward* and *negative attitudes* was, *r* = –.21 and *r* = –.12 in the English-speaking and German sample, respectively. Internal consistencies were acceptable to good for both factors in all samples (Table 5).

### Measurement Invariance Across Gender, Time, and Languages

In Study 1, the highest level of measurement invariance was established (see Table 6), which allows the interpretation of mean differences across gender. In Study 1, men reported higher levels of *positive attitudes* (*p* = .003) and lower levels of *negative attitudes* than women (*p* < .001). In the follow-up of Study 1, again, residual measurement invariance across gender was confirmed. Again, men reported higher levels of *positive attitudes* (*p* = .004) and lower levels of *negative attitudes* than women (*p* < .001). In Studies 2 and 3, measurement invariance across gender could be established, and men reported lower levels of *negative attitudes* than women (*p_english sample_* < .001; *p_German sample_* = .008). We could also establish residual longitudinal measurement invariance for the assessment at the baseline of study one and the follow-up that took place 3 months later, pointing to consistent assessment across time. Stability of manifest scores was *r* = .55, *p* < .001 for *positive attitudes*, and *r* = .60, *p* < .001 for *negative attitudes*. The latent correlations across time were *r* = .62, *p* < .001 and *r* = .68, *p* < .001, respectively. No further model modifications were necessary in any of the models.

**Table 6.** Measurement Invariance of the Two-Factor Solution Between Males and Females and Across Baseline to Follow-Up.

Level of measurement invariance	χ^2^(*df*)	CFI	RMSEA	ΔCFI	ΔRMSEA	Level of measurement invariance	χ^2^(*df*)	CFI	RMSEA	ΔCFI	ΔRMSEA
Study 1: Gender invariance Configural Thresholds Thresholds/loadings Residuals	545 (106) 688 (140) 615 (150) 672 (162)	.980 .980 .979 .977	.088 .077 .076 .077	.000 −.001 −.002	−.011 −.001 .001	Study 2: Gender invariance Configural Thresholds Thresholds/loadings Residuals	336 (106) 378 (140) 396 (150) 426 (162)	.985 .984 .984 .983	.091 .080 .079 .078	−.001 .000 −.001	−.011 −.001 −.001
Follow-up: Gender invariance Configural Thresholds Thresholds/loadings Residuals	803 (106) 833 (140) 846 (150) 867 (162)	.978 .978 .978 .978	.120 .104 .101 .098	.000 .000 .000	−.016 −.003 −.003	Study 3: Gender invariance Configural Thresholds Thresholds/loadings Residuals	234 (106) 251 (116) 255 (150) 282 (162)	.971 .975 .976 .973	.089 .072 .068 .070	.004 .001 −.003	−.017 −.004 .002
Study 1—follow-up: Longitudinal invariance Configural Thresholds Thresholds/loadings Residuals	1529 (234) 1677 (258) 1705 (268) 1790 (292)	.981 .979 .979 .978	.078 .077 .076 .075	−.002 .000 −.001	−.001 −.001 −.001						

*Note.* CFI = comparative fit index; RMSEA = root mean square error of approximation; *df* = degrees of freedom.

### Nomological Network

Table 7 shows the correlations of the two factors with other constructs. In Study 1 and with respect to the cross-sectional assessment, *positive attitudes* correlated positively with appetitive and aversive well-being comparisons (regarding frequency, discrepancy, and affective impact as assessed with the CSS-W). *Positive attitudes* also correlated positively with positive beliefs about worrying (MCQ-30). Meanwhile, *negative attitudes* correlated positively with aversive well-being comparisons (frequency and discrepancy). It further correlated with a more negative affective impact after engaging in aversive and appetitive comparison. Moreover, negative correlations were found with social comparison (SCS), self-esteem (RSES), and age. Positive correlations were found with perceiving worries as uncontrollable (MCQ-30), orientation in life (LOT-R), depression, and anxiety (PHQ-4). Importantly, the two scales of *positive* and *negative attitudes* correlated negatively with one another. Most of the correlations were also found cross-sectionally for the constructs that were assessed at follow-up (i.e., well-being comparisons, metacognitions about worrying, depression, anxiety, and self-esteem; see Table 7).

**Table 7.** Correlations With Validators and Among Each Other.

Study		Study 1		Follow-up		Study 2		Study 3	
Instruments/constructs		Positive-A	Negative-A	Positive-A	Negative-A	Positive-A	Negative-A	Positive-A	Negative-A
ASCI	Positive	—	—	—	—	—	—	—	—
	Negative	−.36*	—	−.20*	—	−.25*	—	−.12	—
CSS-W/A^a^	Aversive frequency	.10*	.42*	.20*	.39*	.25*	.28*	.13	.38*
	Aversive discrepancy	.10*	.39*	.20*	.36*	.25*	.28*	.16*	.37*
	Aversive affect	.10*	−.41*	−.03	−.36*	.01	−.41*	−.09	−.41*
	Appetitive frequency	.21*	.03	.25*	.03	.25*	.01	.18*	.13
	Appetitive discrepancy	.21*	.03	.25*	.02	.23*	−.08	.13	.11
	Appetitive affect	.18*	−.14*	.13*	.12*	.18*	−.22*	.12	−.12
SCS	Social comparisons	.08	−.38*	—	—	—	—	−.11	−.31*
MCQ-30	Positive beliefs	.28*	.04	.31*	.02	—	—	—	—
	Uncontrollability Worrying	−.05	.51*	.08	.48*	—	—	—	—
LOT-R	Orientation in life	−.03	.36*	—	—	—	—	—	—
PHQ-4	Depression	.00	.34*	.01	.42*	.10	.33*	.17*	.35*
	Anxiety	−.03	.39*	.06	.46*	.11	.33*	.11	.40*
RSES	Self-esteem	.04	−.48*	−.04	−.41*	—	—	−.12	−.40*
SMS	Self-enhancement	—	—	—	—	.16*	.01	—	—
	Self-assessment	—	—	—	—	.09	−.07	—	—
	Self-verification	—	—	—	—	.16*	−.14*	—	—
	Self-improvement	—	—	—	—	.15*	.00	—	—
EBQ	Beliefs about emotions	—	—	—	—	.04	.12	—	—
HHTEM	Emotions help	—	—	—	—	.10	.00	—	—
	Emotions hindrance	—	—	—	—	.04	.24*	—	—
SCCS	Self-concept clarity	—	—	—	—	−.12*	−.41*	—	—
BeMaS	Benign envy	—	—	—	—	.33*	−.07	—	—
	Malicious envy	—	—	—	—	.14*	.24*	—	—
Age		−.02	−.11*	−.01	−.11*	−10	−.19*	−.03	−.12

*Note*. A = attitudes; ASCI = Attitudes Toward Social Comparison Inventory; CSS-W/A = Comparison Standards Scale for Well-Being/Appearance; SCS = Social Comparison Scale; MCQ-30 = Meta-Cognitions Questionnaire–30; LOT-R = Life Orientation Test–Revised; PHQ-4 = Patient Health Questionnaire–4; RSES = Rosenberg Self-Esteem Scale; SMS = Self-motives Scale; EBQ = Emotion Beliefs Questionnaire; HHTEM = Help and Hinder Theories about Emotion Measure; SCCS = Self-Concept Clarity Scale; BeMaS = Benign and Malicious Envy Scale.

a The CSS-W was used in all studies expect for the English sample in Study 2 where the CSS-A was used instead.

* *p* < .001.

Concerning the predictive validity of the ASCI, we found that *positive* and *negative attitudes* at baseline predicted the measured constructs at follow-up as follows. Baseline *positive attitudes* correlated positively with follow-up positive beliefs about worrying (MCQ-30; *r* = .21) and appetitive well-being comparisons (CSS-W; frequency: *r* = .22, discrepancy: *r* = .19, affective impact: *r* = .10), but there were no significant correlations with uncontrollability beliefs (MCQ-30), depression and anxiety (PHQ-4), self-esteem, or aversive well-being comparisons (*r*s < .09). Baseline *negative attitudes* correlated positively with follow-up depression and anxiety (PHQ-4 depression: *r* = .33, anxiety: *r* = .41), uncontrollability beliefs (MCQ-30; *r* = .44), and with the frequency and discrepancy of well-being comparisons (CSS-W; frequency: *r* = .36, discrepancy: *r* = .33). They also correlated negatively with follow-up self-esteem (RSES; *r* = –.42) and with the affective impact of aversive well-being comparisons: *r* = –.34). There were no significant correlations with follow-up appetitive comparisons or with positive beliefs about worrying (*r*s < .07).

In Study 2, *positive attitudes* were associated with a higher frequency, discrepancy, and affective impact of appetitive appearance-related comparisons, and with a higher frequency and discrepancy (but not affective impact) of aversive appearance-related comparisons (CSS-A). Positive attitudes also correlated with more pronounced self-motives self-enhancement, self-verification, and self-improvement (SMS); lower self-concept clarity (SCCS); and more benign envy (BeMaS). *Negative attitudes* were correlated with more aversive comparison frequency, discrepancy, and more negative affect (CSS-A), more symptoms of depression and anxiety (PHQ-4), lower self-concept clarity (SCCS), seeing emotions as a hindrance (HHTEM), malicious envy (BeMaS), and younger age.

In Study 3, in the German sample, *positive attitudes* were positively associated with aversive well-being comparison discrepancy, appetitive well-being comparison frequency (CSS-W), and depression (PHQ-4). *Negative attitudes* correlated positively with aversive well-being comparison frequency and discrepancy (CSS-W), depression, and anxiety (PHQ-4) and negatively with aversive well-being affective impact (CSS-W) and self-esteem (RSES).

## Discussion

Given the lack of a scale on individual differences in positive and negative attitudes toward social comparison, we developed and validated the ASCI in three studies, including one longitudinal examination. By employing data-driven reduction methods, we were able to derive a 12-item two-factor solution accounting for *positive* and *negative attitudes toward social comparison*. We confirmed this factor solution in different samples, finding evidence for measurement invariance across gender and time. Furthermore, the scores obtained from the ASCI confirmed many of the anticipated associations with theoretically relevant constructs. Therefore, the ASCI is an economic, reliable, and valid instrument of attitudes toward social comparison that allows to address important research inquiries.

Of our initial item pool that was based on detailed theoretical considerations informed by current literature on comparative thinking (Morina, 2021) as well as existing scales tapping into metacognitions about worrying (Wells & Cartwright-Hatton, 2004), self-evaluation motives (Gregg et al., 2011), and beliefs about emotions (Karnaze & Levine, 2020; Manser et al., 2012), 12 items constituted the final version of the ASCI. Seven of these items reflected the underlying factor *positive attitudes toward social comparison.* This factor contained items tapping into social comparison–oriented self-assessment or decision-making. Individuals may have a variety of reasons to hold positive attitudes toward social comparison depending on their self-motives (Sedikides & Strube, 1997). For instance, this factor correlated with the frequency, discrepancy, and affective impact of appetitive comparisons. This pattern was not restricted to social comparison, but encompassed comparisons to temporal, counterfactual, and criteria-based standards, as captured by the Comparison Standards Scale (Morina & Schlechter, 2023). In addition, our longitudinal analysis revealed that positive attitudes toward social comparison also *predict* engagement in appetitive comparisons concerning their well-being. Positive attitudes toward social comparison were also positively associated with—and predicted—positive beliefs about worrying, suggesting that metacognitions about worries and about social comparison may be closely linked and serve similar cognitive functions. This factor solution and its associated patterns indicate that individuals with positive attitudes toward social comparison may generally use social comparison to obtain information about their social standing (Unkelbach et al., 2023). For example, individuals may use social comparison to evaluate their academic or work-related progress by comparing themselves with peers. Through favorable comparison, individuals may feel better, selectively comparing to downward or lateral social standards and avoiding upward social standards who represent an unattainable end goal. Other individuals may engage in social comparison with the aim of improving their skills or abilities, frequently comparing themselves with individuals who outperform them in relevant dimensions (Sedikides & Strube, 1997). These considerations were supported by moderate correlations with benign envy (Lange & Crusius, 2015) and weaker correlations with the motives self-enhancement, self-verification, and self-improvement (Gregg et al., 2011). Accordingly, this factor can be used to discern a variety of relevant research questions that can shed light on social comparison processes.

The second factor *negative attitudes toward social comparison* consisted of five items and correlated positively with the frequency and discrepancy of aversive well-being and appearance-related comparison. It further correlated with negative affective impact after engaging in well-being comparison. Further correlations were found with unfavorable social comparison, and lower self-esteem, perceiving worries as uncontrollable, lower orientation in life, depression, and anxiety. This indicates that *negative attitudes toward social comparison* are associated with more frequently engaging in comparison in general with a higher perceived discrepancy, and affective impact. In addition, this factor is associated with negative mental health outcomes. This is not surprising given that upward social comparison is related to depression and anxiety (McCarthy & Morina, 2020). Such comparisons are perceived as aversive as they threaten the motives of the comparer, which in turn is associated with lower well-being, depression (Morina & Schlechter, 2023), and self-esteem (Schlechter et al., 2023). Individuals scoring high on this factor may thus see little usefulness in engaging in social comparison and believe that it is detrimental to their well-being and mental health. However, holding negative attitudes toward social comparison may increase the frequency of social comparison by becoming a habitual and uncontrollable behavior, similar to type 2 worries in excessive worry (Wells & Carter, 2001). This notion was supported by high correlations with comparison frequency and metacognitive beliefs about the uncontrollability of worrying and somewhat weaker correlations with emotions as hindrance. Furthermore, this factor was strongly associated with low self-concept clarity indicating that social comparison may pose a threat to individuals with low self-concept clarity (Morina, 2021). The factor negative attitudes toward social comparison enables relevant investigation in psychological research ranging from basic social comparison processes to a deeper understanding of psychopathological mechanisms underlying comparison.

Crucially, the psychometric properties of the scale were robust, as evidenced by good model fit of the two-factor model, excellent factor loadings of the indicators, and internal consistencies of the ASCI scores. Furthermore, the scale demonstrated measurement invariance across gender, which is particularly relevant given that gender differences were observed in the scale, with women exhibiting more negative attitudes toward social comparison compared with men. The demonstration of measurement invariance indicates that gender effects are true effects and not a result of measurement error or other artifacts. These findings are in line with prior research demonstrating that women engage in social comparison more frequently than men and experience greater negative affect following comparison (Morina & Schlechter, 2023), and suggest that attitudes toward comparison may contribute to gender differences. Likewise, ASCI demonstrated measurement invariance across time, which is important for longitudinal investigations. Stability (i.e., retest reliability) of the ASCI scores was only moderate, which is not necessarily surprising as attitudes do not represent stable traits, for which a higher stability would be expected. Given that longitudinal measurement invariance was supported, the moderate stability appears to reflect some genuine changes in attitudes over time. Within 3 months between assessments, individuals may have encountered situations where social comparison was beneficial or harmful to them, and this may have instigated a change in their attitudes. Furthermore, the observed associations with external validators are consistent with the theoretical propositions generated from our theoretical framework both cross-sectionally and longitudinally (Becerra et al., 2020; Gregg et al., 2011; Morina, 2021; Wells & Cartwright-Hatton, 2004), thus supporting convergent and predictive validity of the scale scores. Association with scales that informed our item development was strong for metacognitive beliefs about worrying (Wells & Carter, 2001), but lower correlations were found with self-motives (Gregg et al., 2011) and beliefs about emotions (Becerra et al., 2020). This may reflect the circumstance that self-motives are complex and often not easily obtainable to individuals and are often contingent upon contextual activation (Sedikides & Strube, 1997). However, stronger correlations of ASCI scores with benign envy or self-concept clarity point to the importance of self-motives as they may reflect motives of self-improvement or self-assessment (Sedikides & Strube, 1997). Thus, the present study may not have fully captured the complexity of self-motives, warranting further investigation to better understand the conditions under which individuals engage in upward or downward social comparison. The beliefs about emotions scales were instrumental in developing items that capture attitudes toward social comparison but attitudes concerning emotions may differ from cognitive processes of comparison and worry. Notably, positive and negative attitudes toward social comparison were differentially associated with validators such as positive and negative beliefs about worrying or benign and malicious envy, highlighting the potential of the scale to address nuanced research questions. Nomological networks differed between the German sample and the other samples. In the German sample, positive attitudes were positively associated with depressive symptoms, which we did not find in the other samples. Likewise, the two attitudes toward social comparison factors correlated negatively with each other, but their magnitude differed across studies (range *r*s = –.12 to .42), with the lowest correlation being found in the German study. This may be a random result deriving from the fact that the sample size in the German study was rather low, which needs to be investigated in a replication study with a larger sample. Future studies need to discern whether distinct correlations between samples reflect genuine cross-cultural differences in the adaptive use of social comparison.

Our study has some limitations. Although the study had large sample sizes, it would be prudent for future research to expand the scope of the investigation by using the ASCI with broader, more diverse groups of participants across contexts outside of Western Europe. In addition, the German sample in Study 3 was relatively small, and its gender composition was predominantly female. Therefore, evidence for the validity of the German version of the scale is preliminary at best and further investigation is required. Moreover, we examined the scale properties within the framework of classical test theory. Future research can use item response theory for a more in-depth investigation of item properties or latent reliability (Reise & Waller, 2009). The ASCI exclusively assesses positive and negative attitudes toward social comparison. Although the inclusion of neutral attitudes may provide a comprehensive understanding of the entire spectrum of attitudes toward social comparison, we anticipated limited incremental predictive value in terms of social comparison behavior and other outcomes. Furthermore, we expected that a reasonable amount of the variance associated with neutral attitudes would be captured by the ASCI response options. Yet, future research needs to investigate the role of neutral attitudes toward social comparison and associated outcomes. Our research revealed that negative attitudes exhibited somewhat stronger associations with external validators than positive attitudes toward social comparison. Future research may focus on constructs that should align more closely with positive attitudes toward social comparison, to investigate how such attitudes influence social comparison processes. Subsequently, it would be worthwhile to examine the correlation between ASCI scores and more specific components of comparison processes (e.g., comparison frequencies, standard selection, cognitive, affective, and behavioral responses to comparison outcomes). To allow such an in-depth investigation of positive and negative attitudes and their translational effects, careful experimental settings or experience sampling studies are required. This is an important step to further validate the ASCI, as we validated it against scores of other measures. Given that the construct of attitudes toward social comparison is not empirically established, research needs to examine whether ASCI scores predict actual behavior in naturalistic settings. Moreover, future studies could attempt to thoroughly disentangle different types of social comparison based on their function. For instance, downward social comparison may sometimes serve as a strategic means to counteract negative emotions that arise from upward comparison. In gComp, this constitutes a tertiary comparison that primarily serves the self-enhancement motive by adjusting the consequences of a previous comparison, and is distinct from primary or secondary comparisons that mainly serve self-assessment or self-improvement motives (Morina, 2021). Furthermore, there is currently a lack of knowledge concerning appropriate cut-offs for differences in fit indices to establish measurement invariance in ordinal data. We, therefore, used strict cut-offs, but they need to be interpreted with caution (Chen et al., 2020; Neufeld et al., 2023). Finally, some internal consistencies of the CSS scales were rather low, necessitating careful interpretation of the associations between the ASCI and CSS subscales.

## Conclusion

The ASCI has shown residual measurement invariance across gender and time, and both factors were associated with various relevant constructs. It provides a psychometrically sound tool for researchers and practitioners interested in studying individual differences in attitudes toward social comparison, a pivotal construct in enhancing our comprehension of social comparison processes and their role on individuals’ cognitions, emotions, and behavior.
